# Self-perceived uselessness and associated factors among older adults in China

**DOI:** 10.1186/s12877-016-0406-z

**Published:** 2017-01-09

**Authors:** Yuan Zhao, Jessica M. Sautter, Li Qiu, Danan Gu

**Affiliations:** 1 Ginling College and School of Geography Science, Nanjing Normal University, Nanjing, China; 2 Department of Behavioral and Social Sciences, University of the Sciences, Philadelphia, PA USA; 3 Independent Researcher, New York, NY USA; 4 United Nations Population Division, Two UN Plaza, DC2-1910, New York, NY USA

**Keywords:** Self-perceived uselessness, Self-perception of aging, Usefulness, Successful aging, China, Older adults

## Abstract

**Background:**

Self-perceived uselessness is associated with poor health and high mortality among older adults in China. However, it is unclear which demographic, psychosocial, behavioral and health factors are associated with self-perceived uselessness.

**Methods:**

Data came from four waves (2005, 2008, 2011 and 2014) of the largest nationwide longitudinal survey of the population aged 65 and older in China (26,624 individuals contributed 48,476 observations). This study aimed to systematically investigate factors associated with self-perceived uselessness based on the proposed REHAB framework that includes resources (R), environments (E), health (H), fixed attributes (A) and behaviors (B). Self-perceived uselessness was measured by a single item: “with age, do you feel more useless?” and coded by frequency: high (always and often), moderate (sometimes) and low (seldom and never). Multinomial logistic regression models with low frequency as the reference category were employed to identify REHAB risk factors associated with self-perceived uselessness.

**Results:**

Most factors in the REHAB framework were associated with self-perceived uselessness, although some social environmental factors in the full model were not significant. Specifically, more socioeconomic resources were associated with reduced relative risk ratio (RRR) of high or moderate frequency of self-perceived uselessness relative to low frequency. More environmental family/social support was associated with lower RRR of high frequency of self-perceived uselessness. Cultural factors such as coresidence with children and intergenerational transfer were associated with reduced RRR of high frequency of self-perceived uselessness. Indicators of poor health status such as disability and loneliness were associated with greater RRR of high or moderate frequency of self-perceived uselessness. Fixed attributes of older age and Han ethnicity were associated with increased RRR of high frequency of self-perceived uselessness; whereas optimism and self-control were associated with reduced RRR. Behaviors including regular consumption of alcohol, regular exercise, social participation and leisure activities were associated with reduced RRR of high frequency of self-perceived uselessness.

**Conclusions:**

Self-perceived uselessness was associated with a wide range of factors in the REHAB framework. The findings could have important implications for China to develop and target community health programs to improve self-perceived usefulness among older adults.

## Background

Self-perceived uselessness represents a negative evaluation of one’s usefulness or importance to others and a general understanding about the aging process [1–5]. Self-perceived uselessness, or its opposite, usefulness, is a major component of self-perceived aging: for example, it is one of five items of the Attitude Toward Own Aging subscale of the Philadelphia Geriatrics Center Morale Scale [3]. The feeling of uselessness shapes older adults’ thoughts and behaviors [1–12], which in turn influences psychological and physiological well-being [1, 2, 13].

Empirical studies in both China and Western societies have consistently reported that self-perceived uselessness, a negative self-perception of aging, is a robust predictor of high mortality risk [2, 3, 5, 11, 13–18] and a wide range of poor health indicators such as functional impairment, disability [1–3, 10, 19, 20], chronic conditions [21, 22], lower rates of recovery from illness [23], poorer cognitive and mental health function [20, 24–26], and lower rates of good self-rated health and life satisfaction [20, 27–30]. Studies further indicate that older adults who have higher levels of self-reported uselessness tend to have lower levels of social engagement, physical activity, self-efficacy and self-esteem as well as higher levels of depression [1–4]. Lower levels of self-perceived uselessness with aging are associated with a greater likelihood of survival, better functioning and good life satisfaction [3, 5, 15, 31–34]. These studies have improved our understanding about the significant pathways through which self-perceived uselessness is associated with healthy longevity and successful aging [20].

Researchers have proposed several psychological, physiological and behavioral pathways to explain the possible channels through which self-perceived uselessness affects health and mortality at older ages [18, 20, 34–36]. From a psychological perspective, self-perceived uselessness could diminish beliefs about self-control and self-efficacy that could lead to low resilience capacity and depression, thus preventing psychological well-being [1, 2]. Self-perceived usefulness, by contrast, could lead to a positive appraisal of one’s capacity to deal with adversity or difficulties in daily life [2]. From a physiological perspective, self-perceived uselessness could lead to neuroendocrine and neurohumoral changes, immune alterations, autonomic and cardiovascular dysregulation or central neurotransmitter system dysfunction because of cardiovascular stress [37, 38]. All these could contribute to cardiovascular diseases and subsequent symptoms and disabilities in older age [36, 39]. From a behavioral perspective, attitudes toward aging have the potential to influence responses to illness or physical experiences [31]; self-perceived uselessness could lead to less optimal healthcare seeking behaviors [40] and less engagement in preventive and health-promoting activities [41], subsequently influencing one’s health or leading to more rapid declines in health [35]. On the other hand, positive perceptions of usefulness to families or others would help older adults adapt to age-related changes [42].

One inadequacy of the existing literature is that the majority of research is from non-Western cultures [20, 43, 44]. With a couple of exceptions [18, 20], quantitative research on self-perceived usefulness or uselessness among older adults in China is almost nonexistent; this is primarily due to lack of data on self-perceived uselessness, despite several studies on self-perception of aging [12, 45–47]. It is also unclear whether the risk factors associated with self-perceived uselessness found in Western societies still hold in non-Western nations. It has been argued that different cultures likely have different social views about aging because of different social norms about the social roles of older adults and their role in family systems, which could alter patterns of self-perceived uselessness [48].

The existing literature on self-perceptions of aging and usefulness is also limited by small datasets with a narrow range of age groups and covariates. With a few exceptions [49–51], it is rare to analyze risk factors for the oldest-old. Numerous empirical studies in other areas of aging have shown that the oldest-old aged 80 or older, including centenarians, are likely to have a better capacity to cope with the adversities encountered in daily life [52–56]. Because those who live to advanced ages have had to adapt to many changes and challenges over time, their self-perception of uselessness may differ from that of the young-old aged 65–79 who have experienced fewer challenges. Comparative data from older adults at different levels of longevity may reveal important implications for achieving healthy longevity and successful aging across older ages [20, 52]. Furthermore, most previous studies included relatively small sample sizes, either from local or non-population-based studies [5, 31, 34], which limits the generalizability of the findings.

Finally, almost all existing studies only focus on one or two sets of factors; no studies so far have investigated a wide range of theoretically motivated risk factors from a multidimensional perspective. A more holistic understanding of risk factors would offer a large range of social, demographic, health and behavioral factors to identify older adults who are most likely to need intervention programs to address health problems related to self-perceived uselessness.

Given the power of a single self-rated item like self-perceived uselessness to reflect a wide range of markers related to aging and health, identifying its risk factors may have important implications for public health surveillance and health services research aimed at achieving successful aging and healthy longevity [20]. A growing body of research has investigated factors associated with self-perceived uselessness and aging, as reviewed above, but there are several ways that new research can add to this literature.

To extend existing research in healthy longevity, this study aims to investigate which socioeconomic resources, social environments, health statuses, fixed attributes and health behaviors are associated with self-perceived uselessness among older adults in mainland China (hereafter China). Data come from the Chinese Longitudinal Healthy Longevity Survey (CLHLS), the largest ongoing nationally-representative sample and the only nationwide survey in China that collects data on self-perceived uselessness in addition to demographics, resources, environmental factors and health status. The focus on Chinese older adults has profound significance. In contemporary China, around 20% of adults aged 65 years or older, more than 25 million older adults, feel useless always or often [20]; about 50–70% of older adults reported feelings of being a family burden, getting older and falling behind social progress [20]. This large population of older adults with a negative perception of usefulness is likely to experience higher mortality [18], higher risk of disability and cognitive impairment [20], and higher prevalence of depression and loneliness [56, 57]. Self-perceived uselessness is becoming a public health challenge for China. A systematic investigation of factors that may be closely linked with self-perceived uselessness at older ages would help to identify risk factors and target appropriate interventions for subpopulations at highest risk.

In the next section, we provide a brief review of risk factors for feeling useless at older ages, organized with a new conceptual framework that guides the present study.

### Factors associated with uselessness and the REHAB framework

The existing literature on factors associated with self-perceived uselessness is very limited. However, there have been quite a few studies that have examined factors associated with self-perception of aging [47, 48, 58]. Because self-perceived uselessness is a key component of self-perceived aging, our review includes both self-perceived uselessness and self-perceived aging [3, 10].

**Fig. 1:**
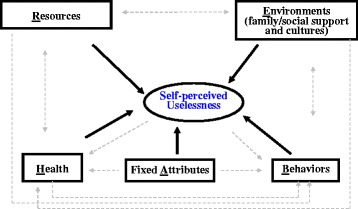
Conceptual framework for the multidimensional study of self-perceived uselessness. Note: The underlined letter of each set of factors was used to name the framework: REHAB. Bold solid arrows represent possible linkages under study, while grey dashed arrows represent possible linkages beyond the scope of this study

Overall, empirical studies have shown that a number of factors are independently associated with self-perceptions of uselessness or aging [45, 48, 58]. We classified these factors as resources (R), environments (E), health (H), fixed attributes (A) and behaviors (B). Resource factors mainly include socioeconomic status (SES); environmental factors mostly refer to social environments that include family/social supports and cultural factors; health conditions could include various indicators measuring different dimensions of health; fixed attributes mainly include age, gender, ethnicity, predisposition and some biological components; and behavioral or lifestyle factors usually consist of smoking, drinking, involvement in leisure activities and social participation. Accordingly, we propose a conceptual framework named REHAB to systemically examine how these sets of factors are associated with self-perceived uselessness. We follow a conventional approach in the literature and begin with fixed attributes (mainly demographics) (Fig. 1).

### Fixed attributes (A)

Most studies have revealed that, among older adults in various populations, increasing age is associated with more negative perceptions of aging and uselessness [47, 49, 59–61]. However, several studies have found that age is not associated with self-perception of aging [58, 62], even when health conditions are taken into account [63]. Gender differences are also inconclusive. Some studies have found that men tend to have a more positive perception about their own aging than women [58, 64], while others have found opposite results [65], and still others have found no gender differences [49–52, 59]. Racial differences in self-perception of aging are well-documented, but such differences are largely due to cultural practices and norms [66]. Individual predispositions such as optimism and self-control may help develop good skills to cope with daily challenges and promote social engagement [67]. Both optimism and self-control are associated with positive perceptions of aging and usefulness [64, 68].

### Resources (R)

One’s self-perception of aging is contingent upon socioeconomic resources available to that person [68]. Studies have shown that lack of resources could lead to a negative self-perception about aging, while adequate or sufficient resources could lead to positive perceptions about aging [67]. This is because older adults with more resources have more opportunities to be involved in various social connections and feel useful to others. Wealthier people are also likely to feel more excited and hopeful about their lives ahead [69]. However, some studies have found no differences by resources such as education [47, 70]; others have found that higher income and educational attainment are associated with less positive self-perceptions of aging because of relative losses perceived after retirement [47, 59, 70]. Access to other resources such as greater medical care tended to be associated with more positive perceptions about aging [61]. Additional studies have revealed that there is a negative association between neighborhood-level socioeconomic development and self-perception of aging in more advanced societies due to increased individual independence and weakened multi-generational family structure that develop with industrialization and modernization [45, 71]. The socioeconomic resources of family members and significant others are also important factors influencing one’s own resources, physical health and quality of life [72, 73].

### Environments (E)

Social environments include family/social support and cultural conditions. The individual assessment of one’s usefulness to others at older ages is a social process that reflects the internalization of culturally appropriate attributes [74]. This social process could be influenced by family members that either reinforce or challenge previous perceptions, thus affecting self-perceived aging or usefulness [75].

#### Social support

Social relations with family and friends are a central source of social support in later adulthood [58]. Self-perceptions of aging and usefulness may be influenced by social comparisons with network members (relatives, friends and neighbors) surrounding older adults [46]. The existence of strong social ties and support from others may bolster older individuals’ self-esteem, positively influence their self-perception of aging and health [67], and make people aware of positive age-related changes [76]. Older adults who are socially connected generally report more positive feelings about their aging process [77].

The contact hypothesis posits that social contact and interactions could lead to a reduction in negative perceptions of aging and uselessness through improved communication and interaction with members in the network [78]. Studies have shown that fewer social ties and low frequency of interactions are associated with increased perceptions of uselessness [2, 14, 71, 76]. For older men, marriage is an important basis of social support, with spouses both sustaining health behaviors and facilitating physical care, especially when there is a reduction in network size of family and friends [67]. The socioemotional selectivity theory argues that social network sizes may decline in later ages, but family ties remain important as older adults shift their focus to more emotionally meaningful intimate relationships (i.e., family members and close friends) [1, 79]. However, when social support includes personal care, the receipt of care services from spouses, children, family members or friends could increase negative self-perceptions of aging through intensified feelings of dependence on others, which implies a loss of control and burden [80]. Studies on the association between social services and self-perception of aging are almost nonexistent.

#### Culture

Cultural meanings are essential for self-perception of aging or usefulness [58]. Identity theory emphasizes the influence of society on individuals [78]. Because cultural systems shape one’s views about aging [80–82], self-perception of aging is a product of societal beliefs [5] that differ across cultures [58, 64, 82]. Scholars have argued that Eastern cultures emphasize respect to one’s elders [50, 76]; for example, societies influenced by Confucian values and the practice of filial piety promote positive views of aging and usefulness in old age [50, 53, 83–85]. In contrast, Western societies hold more negative views about the aging process due to youth-oriented value systems [45, 58, 82, 84, 85]. Consequently, self-perceptions of aging are more positive in Confucian countries like China compared to Western cultures [45, 84]. However, the societal attitude toward older adults in China is changing because of industrialization and rapid population aging [48].

### Behaviors (B)

There is a consensus that healthy behaviors such as frequent participation in leisure activities, exercise and social engagements could lead to positive perceptions of aging, whereas low participation and inactivity may erode feelings of usefulness [47, 48]. This is because activities imply regular commitments, membership, identity and integration [58]. Social engagements may also stimulate multiple body functions (e.g., cognitive, cardiovascular, neuromuscular), protect against cognitive decline [86], bolster active coping strategies, and, lower the risk of mortality. These activities thus can be important contributors to feelings of meaningfulness, purposefulness and usefulness; in turn, these feelings can reinforce individuals’ desires to maintain social connections and engagement [1]. Regular involvements in leisure and physical activities at late ages could buffer against the negative impacts of mishaps, age-related physical changes and life events, and provide opportunities to successfully cope with these challenges and adversities in daily life [34, 58]. Meaningful social roles for older adults could promote the image of older adults at the societal level [58]. On the other hand, no participation in leisure and social activities could cause increased feelings of loneliness, isolation, abandonment, distress and negative perception of aging.

### Health (H)

Health can be considered the most important element in the self-assessment of aging and usefulness [5, 45, 58, 84]. Declines in functioning and health status may prohibit older adults from providing meaningful services to others, and thus negatively impact perceptions about their level of usefulness [2]; better physical health (few chronic conditions, no functional disability) can be associated with more positive feelings about aging [77]. One recent study revealed that the presence of various health problems (in terms of chronic conditions, poor functioning and greater disability) was associated with more negative perceptions of aging or uselessness [67]. Evidence further shows that physical health may play a more central role in self-perceptions of aging than cognitive function [45]. Psychological well-being could reduce disease, disability and mortality through protective behaviors and thus eventually improve positive perceptions of aging [58].

## Methods

### Study sample

We pooled four waves of the Chinese Longitudinal Healthy Longevity Survey (CLHLS) in 2005, 2008–2009, 2011–2012 and 2014 to increase the sample size to obtain more reliable results. The pooled datasets were constructed longitudinally, similar to some recent studies [20]. Three waves in 1998, 2000 and the 2002 were not included in this analysis because many important variables were not available. The CLHLS is conducted in a randomly selected half of the counties/cities in 22 provinces where Han is the majority ethnicity. Nine predominately minority provinces were excluded to avoid inaccuracy of age-reporting at very old ages (e.g., ages 90+) among minorities [87]. The total population of these 22 provinces accounted for 82% of the total population of China in 2010.

The analytical sample for this study consisted of 26,624 respondents who contributed 48,476 observations from 2005 to 2014. The sampling procedures and assessments of data quality of the CLHLS can be found elsewhere and thus are not detailed here [20, 87].

### Measurements

#### Self-perceived uselessness

The CLHLS designed a single question to collect data on self-perceived uselessness: “As you age, do you feel more useless?” The wording is almost identical to the wording of the “As you get older, you are less useful” item in the Attitude Toward Own Aging subscale of the Philadelphia Geriatrics Center Morale Scale [3, 10]. There are six response categories for self-perceived uselessness based on frequency: always, often, sometimes, seldom, almost never or never and unable to answer. To obtain more reliable results, we reclassified them into three levels of frequency plus one special category: always/often (high frequency), sometimes (moderate frequency), seldom/never (low frequency) and unable to answer. The main purpose of keeping “unable to answer” as a response category was to keep original information intact and to better reflect true associations with levels of self-perception, including being unable to assess due to poor health. Of the participants who selected “unable to answer,” about 90% were unable to answer due to poor health [20].

#### Factors associated with self-perceived uselessness

Based on the REHAB framework proposed above, we modeled the following six sets of factors to examine whether they are associated with self-perceived uselessness: resources (R), environments (E), health conditions (H), fixed attributes (A) and behaviors (B).

The fixed attributes (A) included age, sex (men vs. women), ethnicity (Han vs. non-Han) and two predisposition variables. The variable age (in years) was grouped into 65–79, 80–89, 90–99 and 100+. Optimism was measured by the question “do you look on the bright side of things?” and self-control was measured by the question “do you have control over the things that happen to you?”. Both predisposition variables have six response categories: always, often, sometimes, seldom, never and not able to answer. We combined always and often into one category (high), and combined sometimes, seldom and never into another category (low). For the respondents who were not able to answer the questions, we imputed them into one of the five categories by assuming that their answers would be the same as those who answered the question if they had the same demographics, resources, family/social support, behaviors and health conditions.

Resources (R) were mainly measured by the respondent’s socioeconomic status (SES) that included residence (urban vs. rural), years of schooling (0, 1–6 and 7+), lifetime primary occupation (white collar occupation vs. others), economic independence (having a retirement wage/pension and/or own earnings vs. no), and family economic conditions (rich vs. fair/poor). Education of other family members, including years of schooling of spouse (0, 1–6, 7+ and missing/no spouse), coresident children/grandchildren (0, 1–6, 7–9, 10+ and missing/no children/grandchildren), and father (0, 1+ and missing) were also considered as SES factors. Around 15-40% of the respondents did not provide information for educational attainment levels of other family members because they could not remember or the question was not applicable (e.g., no coresident children/grandchildren, never married), so we kept a category of missing to fully reflect the data. Considering urban–rural residence as an SES factor is a common practice in China due to significant rural–urban differences in economic development [88].

Social environmental factors (E) were measured by family/social support and cultural context. The former included marital status (currently married vs. no), most frequently contacted person (family member, friend/relative and nobody), most trusted person (family member, friend/relative and nobody), most helpful person (family member, friend/relative and nobody), availability of community-based care services in the neighborhood (yes vs. no), and availability of community-based social activities and entertainment services in the neighborhood (yes vs. no). Proxy factors for culture included coresidence with children (yes vs. no) and match between expected living arrangements (coresidence with children, living alone or with spouse only, and institutionalization) and actual living arrangements (concordance vs. discordance). Other measures of culturally expected support include receiving financial and instrumental support (money or food) from children (yes vs. no), and giving financial and instrumental support to children (yes vs. no). In the literature on aging and social gerontology, coresidence has been used either as a proxy of social connectedness and social support [89] or as a cultural tradition [90–96]. Many studies argue that the high prevalence of coresidence with adult children among older parents in China and other East Asian countries is mainly due to the long history of Confucianism [97]. In the present study, we considered coresidence as a cultural tradition.

Behavioral factors (B) were measured by currently smoking (yes vs. no), currently consuming alcohol (yes vs. no), regularly exercising (yes vs. no), and frequency of leisure activities and social participation. Levels of leisure activities were constructed from the sum of frequencies of six items, including doing housework, gardening, raising domestic animals or poultry, reading books/newspapers, watching TV/listening to radio and any other personal outdoor activities. Each item was measured on a five-point Likert-scale from never to almost daily. The reliability coefficient of these seven items is 0.66. The tertile was applied to classify the sample into three equal-sized groups: low level, moderate level and high level of leisure activity. Social participation was measured by two questions “do you participate in social activities?” and “do you play cards/mah-jong?”. We similarly classified the sample into three groups: low level (never involved in these two activities), high level (involved in one of the two activities 1–7 times per week), and moderate level (the rest of the sample).

Health conditions (H) included activities of daily living (ADL) disability, instrumental activities of daily living (IADL) disability, cognitive function, chronic disease conditions and subjective wellbeing. ADL disability was measured by self-reported ability to perform six daily activities (bathing, dressing, indoor transferring, toileting, eating and continence). Following the common practice in the field [18], we classified the respondents into two groups: needing assistance in any one of the six tasks (ADL dependent/disabled) versus needing no assistance in any of the six tasks (ADL independent/not-disabled). IADL was measured by self-reported ability to perform eight activities: (a) visiting neighbors, (b) shopping, (c) cooking, (d) washing clothes, (e) walking one kilometer, (f) lifting 5 kg, (g) crouching and standing up three times, and (h) taking public transportation. In a similar vein, we dichotomized the respondents into two groups: needing help in performing any of these eight IADL items (IADL disabled/dependent) versus needing no help in performing any of the eight activities (IADL not-disabled/independent). Cognitive function was measured by a validated Chinese version of the Mini-mental State Examination (MMSE), which included six domains of cognition (orientation, reaction, calculation, short memory, naming and language) with a total score of 30 [87]. We dichotomized the respondents into impaired (scores <24) and unimpaired (scores 24–30) based on the cut-point commonly used in aging research [87]. An alternative cut-point score (18) was also examined and yielded very similar results. Chronic disease condition was dichotomized into whether the respondent reported any disease at the time of survey from a list of more than twenty conditions (hypertension, heart diseases, stroke, diabetes, cancer, etc.) versus none. Fewer than 5% of the respondents had 2+ conditions and the prevalence of disease conditions was comparable to those found in other nationwide surveys [87]. Subjective (psychological) wellbeing was measured by two variables: “do you feel lonely?” (loneliness), and “do you feel as happy as you did when you were younger?” (joyfulness). Scoring for these variables is identical to optimism and self-control, the two predisposition variables (high vs. low).

#### Analytical strategy

Because the outcome variable of self-perceived uselessness included four categories (high frequency, moderate frequency, low frequency and unable to answer), multinomial logistic regression models were employed to examine what factors were associated with frequency of self-perceived uselessness compared to the low level (reference group). The results were reported as a relative risk ratio (RRR) [98]. Results for “unable to answer” were not presented to better focus on the research objectives. In order to obtain more robust and reliable results, we pooled all four waves of the data together and adjusted for intrapersonal correlation across waves. Seven different models were analyzed, including six models for each individual set of factors (two models for environmental factors) and one full model that included all sets of study factors. Because fixed attributes include demographics that are the most basic characteristics of respondents, and because there are substantial differences in health and resources between demographic groups [87], fixed attributes were included in all seven models. A variable reflecting survey year was also included in all models to account for possible trends over time.

With few exceptions that we noted above (i.e., educational attainments of spouse and father, two fixed attributes and two subjective wellbeing variables), the proportions of missing values for other variables under study were less than 2%. To minimize biases, we used multiple imputation techniques to impute these missing values although the mode of each categorical variable produced very similar results. Sampling weights were not applied in the regression analysis because the CLHLS weight variable does not reflect the national population distributions with respect to variables other than age, sex and urban or rural residence [99]. Weighted regressions could unnecessarily enlarge standard errors [100], so we chose to present the unweighted regression models that produce unbiased coefficients when including variables related to sample selection (i.e., age, sex and urbanicity) [101]. We found that multicollinearity among variables was not a problem, with all variance inflation factors less than 3 [102, 103]. All analyses were performed using Stata version 13.1 [98].

## Results

### Prevalence of self-perceived uselessness

Table 1 lists the distributions of conceptual framework factors in the pooled sample and the prevalence of self-perceived uselessness categories for the total sample and for each level of the conceptual framework factors. The distributions in Table 1 are based on 48,476 observations from 26,624 individuals. The percentage distributions in the table were derived from all observations, although the distributions were similar if they were based on the number of respondents. In the sample, low frequency of self-perceived uselessness was most prevalent (33.0%), followed by moderate frequency (31.2%), and high frequency (23.0%). About 12.8% were not able to answer the question. The weighted distribution of self-perceived uselessness was 19.2% for high frequency, 34.0% for moderate frequency, 43.8% for low frequency, and 3.0% for unable to answer (not shown). These weighted estimates suggest that about one-fifth of older adults in contemporary China often or always feel useless. The weighted percentage for high frequency was 22% for women and 16% for men.

**Table 1:** Distribution of the pooled sample: 2005, 2008, 2011 and 2014 waves of the CLHLS

		Self-perceived uselessness (percentage)			
Variables	Sample %^a^	Always/often	Sometimes	Seldom/never	Unable to answer
Total sample	48,476 (100%)	11,147 (23.0%)^b^	15,122 (31.2%)^b^	16,000 (33.0%)^b^	6207 (12.8%)^b^
Resources (R)					
Own education, 0 years of schooling	66.0	24.9	30.7	28.5	15.9
Own education, 1–6 years of schooling	25.1	20.8	32.9	38.7	7.6
Own education, 7+ years of schooling	8.9	15.0	29.9	50.2	4.9
Spouse’s education, 0 years of schooling	54.3	24.5	30.9	30.0	14.7
Spouse’s education, 1–6 years of schooling	21.4	22.8	31.6	36.1	9.5
Spouse’s education, 7+ years of schooling	7.4	17.6	30.2	45.5	6.7
Spouse’s education, missing or not applicable	16.9	20.8	32.0	33.5	13.7
Father’s education, 0 years of schooling	69.3	24.5	31.0	31.2	13.3
Father’s education, 1+ year of schooling	15.4	19.8	32.5	40.6	7.1
Father’s education missing	15.3	19.7	30.8	33.4	16.2
Rural	56.4	24.7	31.4	30.2	13.8
Urban	43.6	20.9	30.9	36.7	11.6
Non-white collar occupation	92.3	23.7	31.3	31.7	13.3
White collar occupation	7.7	14.2	29.7	49.2	6.9
Economic dependence	72.2	25.2	30.7	28.4	15.7
Economic independence	27.8	17.3	32.5	45.0	5.2
Fair or poor family economic condition	84.8	24.1	31.5	31.0	13.4
Rich family economic condition	15.2	16.8	29.3	44.5	9.3
Environments (E) -- Family/social support					
Currently not married	66.0	24.5	30.2	28.2	17.1
Currently married	34.0	20.1	33.1	42.3	4.5
Most frequently contacted person - family member	77.0	22.2	32.5	34.6	10.8
Most frequently contacted person – friend/relative	16.9	26.9	32.1	33.6	7.4
No one to contact	6.1	24.3	15.1	14.8	45.8
Most trusted person - family member	87.8	22.7	32.3	34.2	10.9
Most trusted person – friend/relative	6.6	27.4	30.9	31.9	9.8
No one to trust	6.6	25.4	18.4	18.5	37.8
Most helpful person - family member	92.5	23.1	31.7	33.7	11.5
Most helpful person – friend/relative	4.8	28.2	29.5	29.4	12.9
No one to ask for help	2.7	24.7	18.2	18.7	38.4
Community-based care services available - no	95.5	23.0	31.3	32.8	12.8
Community-based care services available - yes	4.5	22.8	28.0	36.7	12.5
Community-based social services available - no	88.0	23.4	31.0	32.2	13.4
Community-based social services available - yes	12.0	19.9	32.6	39.2	8.3
Environments (E) --Cultural tradition					
Coresidence with children - no	39.3	24.3	32.5	35.6	7.6
Coresidence with children - yes	60.7	22.2	30.4	31.3	16.2
Discordant living arrangements/institutionalization	26.3	22.3	27.5	30.0	20.2
Concordance in living alone or with spouse	27.4	24.4	33.7	37.9	4.0
Concordance in coresidence with children	46.3	22.6	31.8	31.8	13.8
Receiving money/food from children - no	20.0	21.5	28.5	33.5	16.6
Receiving money/food from children - yes	80.0	23.4	31.9	32.9	11.9
Giving money/food to children - yes	77.0	24.5	30.2	30.2	15.1
Giving money/food to children - no	23.0	18.1	34.5	42.5	5.0
Health conditions (H)					
ADL independent	74.6	21.8	33.7	38.1	6.5
ADL dependent	25.4	26.4	24.0	18.2	31.5
IADL independent	32.0	15.5	33.1	49.7	1.7
IADL dependent	68.0	26.5	30.3	25.2	18.0
Cognitively unimpaired	60.1	21.1	35.7	41.7	1.4
Cognitively impaired	39.9	25.8	24.4	19.9	30.0
No chronic disease	40.1	19.6	31.1	36.6	12.7
1+ chronic disease	59.9	25.2	31.3	30.6	12.9
Low joyfulness	64.2	25.8	33.7	25.4	15.1
High joyfulness	35.8	18.1	26.8	46.5	8.6
Low loneliness	92.9	19.9	31.8	34.8	13.5
High loneliness	7.1	62.9	23.3	10.1	3.8
Fixed attributes (A)					
Mean age (years)	86.4^c^	--	--	--	--
Age 65–79	30.3	19.7	34.3	43.8	2.3
Age 80–89	26.7	26.4	33.3	32.7	7.6
Age 90–99	26.0	24.7	29.9	28.1	17.3
Age 100+	17.0	20.9	24.4	21.9	32.8
Female	56.4	25.0	30.2	28.8	16.0
Male	43.6	20.4	32.5	38.4	8.7
Non-Han ethnicity	16.3	19.3	34.0	34.2	12.5
Han ethnicity	83.7	23.7	30.7	32.8	12.9
Low optimism	24.0	33.1	32.7	19.1	15.1
High optimism	76.0	19.8	30.7	37.4	12.1
Low self-control	46.1	24.3	30.5	24.8	20.3
High self-control	53.9	21.9	31.8	40.0	6.4
Behaviors (B)					
Currently smoking - no	82.3	23.5	30.8	31.8	14.0
Currently smoking - yes	17.7	20.8	33.2	38.8	7.1
Currently consuming alcohol - no	82.4	23.8	31.0	31.5	13.7
Current consuming alcohol - yes	17.6	19.2	32.0	40.2	8.6
Regularly exercising - no	70.8	25.2	31.0	27.8	16.0
Regularly exercising - yes	29.2	17.6	31.7	45.7	5.0
Leisure activity low level	33.0	26.7	24.5	19.1	29.7
Leisure activity medium level	31.2	25.1	34.5	33.5	7.0
Leisure activity high level	35.6	17.8	34.5	45.4	2.4
Social participation low level	75.6	25.2	30.6	28.5	15.7
Social participation medium level	11.0	16.7	34.6	43.4	5.4
Social participation high level	13.6	15.8	31.6	49.8	2.8
Survey years					
Wave 2005	31.6	23.2	32.2	33.4	11.3
Wave 2008	33.7	23.9	29.1	31.9	15.1
Wave 2011	20.0	22.1	31.9	34.4	11.6
Wave 2014	14.7	21.7	33.0	33.0	12.3

Note: (1) Except for the sample size in the top line, all numbers were percentages unless otherwise stated. (2)^a^ this column referred to percentage distribution of each category of the study variables among 48,476 observations from 26,624 individuals who were interviewed from 2005 to 2014. The distributions by 26,624 individuals at their baseline were similar to what were presented in the table. (3)^b^ percentages of self-perceived uselessness were calculated by row. The row sum of percentage of self-perceived uselessness may not be equal to 100% due to roundness. (4)^c^ mean age. (5) The results were unweighted. (6) --, not applicable

### Factors associated with self-perceived uselessness

Tables 2 and 3 present relative risk ratios (RRR) from multinomial logistic regression models of REHAB factors associated with high frequency and moderate frequency of self-perceived uselessness relative to low frequency. We summarize several major findings below.

**Table 2:** Factors associated with high vs. low frequency of self-perceived uselessness, CLHLS 2005–2014

	High frequency vs. low frequency of self-perceived uselessness						
	Model I	Model II	Model III	Model IV	Model V	Model VI	Model VII
Fixed attributes (A)							
Age 80–89 (age 65–79)	1.69***	1.49***	1.55***	1.65***	1.38***	1.15***	1.04
Age 90–99 (age 65–79)	1.76***	1.51***	1.51***	1.74***	1.21***	0.92+	0.80***
Age 100+ (age 65–79)	1.76***	1.45***	1.51***	1.77***	1.07	0.78***	0.66***
Male (female)	0.70***	0.82***	0.74***	0.70***	0.78***	0.82***	0.95
Han (non-Han)	1.44***	1.48***	1.54***	1.44***	1.53***	1.38***	1.41***
High optimism (low)	0.33***	0.35***	0.34***	0.33***	0.36***	0.48***	0.52***
High self-control (low)	0.73***	0.80***	0.71***	0.73***	0.86***	0.86***	0.89**
Resources (R)							
1–6 years of schooling (0)		0.84***					0.92*
7+ years of schooling (0)		0.69***					0.77***
Spouse 1–6 years of schooling (0)		0.96					1.01
Spouse 7+ years of schooling (0)		0.85*					0.91
Spouse education missing (0)		0.92					0.83*
Father 1+ years of schooling (0)		0.88**					0.92+
Father education missing (0)		0.90+					0.88+
Urban (rural)		0.86***					0.85***
White collar (no)		0.88*					0.83**
Economic independence (dependence)		0.70***					0.80***
Rich family economic condition (fair/poor)		0.62***					0.77***
Environments (E) -- Family/social support							
Currently married (no)			0.82***				0.98
Most frequently contacted person, friend/relative (family member)			1.19**				1.23***
No one to contact (family member)			1.80***				1.16+
Most trusted person, friend/relative (family member)			0.94				0.91
No one to trust (family member)			1.17+				1.06
Most helpful person, friend/relative (family member)			1.26**				1.04
No one to ask for help (family member)			1.22+				1.15
Community care services available (no)			0.90				0.89
Social services available (no)			0.76***				0.98
Environments (E) --Cultural tradition							
Coresidence with children (no)				0.91+			1.06
Concordant living alone/with spouse (discord.)				1.01			1.28***
Concordant coresid. with children (discord.)				0.89**			0.88***
Receiving money or food from children (no)				1.11***			1.03
Giving money or food to children (no)				0.62***			0.83***
Behaviors (B)							
Currently smoking (no)					1.09*		1.06
Currently consuming alcohol (no)					0.83***		0.87**
Regular exercise (no)					0.65***		0.72***
Leisure activity medium level (low)					0.70***		0.90**
Leisure activity high level (low)					0.52***		0.84***
Social participation medium level (low)					0.65***		0.76**
Social participation high level (low)					0.63***		0.75***
Health conditions (H)							
ADL disabled (no)						1.53***	1.64***
IADL disabled (no)						2.20***	1.96***
Cognitively impaired (no)						1.44***	1.22***
1+ chronic conditions (none)						1.37***	1.47***
High joyfulness (low)						0.50***	0.55***
High loneliness (low)						8.02***	7.42***
Wave 2008 (2005)	1.06*	1.03	1.06	1.05	1.04	0.96	0.93
Wave 2011 (2005)	0.97	0.97	0.98	0.97	0.96	1.04	1.03
Wave 2014 (2005)	1.22***	1.25***	1.52***	1.25**	1.21***	1.24***	1.72***

Note: (1) Factors in this Table were not organized by the order of letter of the REHAB framework. (2) Figures in the table were relative risk ratios based on multinomial logit regression. (3) High frequency of feelings of uselessness referred to always/often and low frequency referred to seldom/never. Results for “unable to answer” and moderate frequency were not presented. (4) Category in the parentheses of each variable was the reference group. (5) + *p* < 0.1, *p < 0.05, **p < 0.01, ***p < 0.001

**Table 3:** Factors associated with moderate vs. low frequency of self-perceived uselessness, CLHLS 2005–2014

	Moderate frequency vs. low frequency of self-perceived uselessness						
	Model I	Model II	Model III	Model IV	Model V	Model VI	Model VII
Fixed attributes (A)							
Age 80–89 (age 65–79)	1.24***	1.17***	1.18***	1.23***	1.17***	1.05	1.00
Age 90–99 (age 65–79)	1.27***	1.18***	1.15***	1.25***	1.13***	0.96	0.89**
Age 100+ (age 65–79)	1.27***	1.16***	1.14**	1.26***	1.08+	0.90*	0.82***
Male (female)	0.87***	0.94*	0.92***	0.88***	0.92***	0.93***	1.02
Han (non-Han)	1.01	0.99	1.05	1.01	1.05	1.02	1.06
High optimism (low)	0.51***	0.53***	0.50***	0.51***	0.53***	0.63***	0.64***
High self-control (low)	0.74***	0.77***	0.73***	0.73***	0.79***	0.85**	0.86***
Resources (R)							
1–6 years of schooling (0)		0.93*					0.95
7+ years of schooling (0)		0.79***					0.83**
Spouse 1–6 years of schooling (0)		0.95					0.98
Spouse 7+ years of schooling (0)		0.89*					0.92
Spouse education missing (0)		0.92					0.90
Father 1+ years of schooling (0)		0.99					1.00
Father education missing (0)		0.87**					0.90+
Urban (rural)		0.92**					0.92**
White collar (no)		0.94					0.92+
Economic independence (dependence)		0.88***					0.92*
Rich family economic condition (fair/poor)		0.74***					0.82***
Environments (E) -- Family/social support							
Currently married (no)			0.84***				0.89**
Most frequently contacted person, friend/relative (family member)			1.03				1.08*
No one to contact (family member)			0.99				0.86*
Most trusted person, friend/relative (family member)			0.92				0.90
No one to trust (family member)			0.93				0.91
Most helpful person, friend/relative (family member)			1.12				1.04
No one to ask for help (family member)			1.02				1.05
Community care services available (no)			0.81**				0.81**
Community social services available (no)			0.94				1.08*
Environments (E) --Cultural tradition							
Coresidence with children (no)				0.90*			0.93
Concordant living alone/with spouse (discord.)				1.02			1.09*
Concordant coresid. with children (discord.)				1.06+			1.02
Receiving money or food from children (no)				1.13***			1.02
Giving money or food to children (no)				0.88***			1.01
Behaviors (B)							
Currently smoking (no)					1.07*		1.05+
Currently alcoholic consuming (no)					0.90**		0.94+
Regular exercise (no)					0.75***		0.81***
Leisure activity medium level (low)					0.97		1.05
Leisure activity high level (low)					0.89**		1.08+
Social participation medium level (low)					0.88***		0.94+
Social participation high level (low)					0.75***		0.82***
Health conditions (H)							
ADL disabled (no)						1.12***	1.21***
IADL disabled (no)						1.57***	1.52***
Cognitively impaired (no)						1.03	0.97
1+ chronic conditions (none)						1.14***	1.19***
High joyfulness (low)						0.47***	0.50^****^
High loneliness (low)						2.08***	2.07***
Wave 2008 (2005)	0.94*	0.92**	0.95*	0.94**	0.92**	0.82*	0.82*
Wave 2011 (2005)	0.98	0.99	0.99	0.99	0.98	1.05	1.05
Wave 2014 (2005)	1.07	1.15*	1.16**	1.09*	1.07	1.12**	1.32***

Note: (1) Factors in this Table were not organized by the order of letter of the REHAB framework. (2) Figures in the table were relative risk ratios based on multinomial logit regression. (3) Moderate frequency referred to sometimes and low frequency referred to seldom/never. Results for “unable to answer” and high frequency were not presented. (4) Category in the parentheses of each variable was the reference group. (5) + *p* < 0.1,*p < 0.05, **p < 0.01,***p < 0.001

#### Fixed attributes were strongly and consistently associated with self-perceived uselessness

Model I in Table 2 shows that all fixed attribute factors are associated with risk of high frequency of self-perceived uselessness. Compared to younger ages 65–79, octogenarians (ages 80–89), nonagenarians (ages 90–99) and centenarians (ages 100+) experienced increased risk of high frequency of self-perceived uselessness relative to low frequency by 69, 76 and 76%, respectively. These risk ratios were slightly attenuated in Models II through IV when resources and environmental factors were taken into account. However, when behavioral factors were considered (Model V), these risk ratios were substantially reduced and non-significant for the centenarian group. Interestingly, when health conditions were considered in the analysis (Model VI), octogenarians and centenarians tended to have 8 and 22% lower RRR for high frequency of self-perceived uselessness, respectively; these results were even more pronounced in the full model, with reduced risks of 20% for nonagenarians and 35% for centenarians compared to young-old adults aged 65–79 years old (Model VII). The reduced risk at oldest ages, independent of health statuses and health behaviors, was similar but weaker for moderate frequency versus low frequency (Table 3).

Male gender was associated with 18–30% lower RRR for high frequency of self-perceived uselessness relative to low frequency, compared to women, when each set of factors was added individually (Models I to VI). However, no gender difference was found when all sets of factors were simultaneously included in the model (Model VII). Results for moderate frequency versus low frequency in Table 3 were similar despite reduced RRRs. Participants of Han ethnicity tended to have 38–54% greater RRR for high frequency of self-perceived uselessness relative to low frequency, compared to minority ethnicity (Table 2); no ethnic difference was found for moderate frequency versus low frequency (Table 3). High level of optimism and self-control were associated with 48–66% and 11–29% lower RRR for high frequency relative to low frequency of self-perceived uselessness, respectively (Table 2), although their RRRs were reduced when comparing moderate frequency with low frequency (Table 3).

#### People with more resources tend to report low frequency of self-perceived uselessness

Model II in Table 2 shows that more socioeconomic resources were associated with lower RRR for high frequency of self-perceived uselessness relative to low frequency. Specifically, compared to the zero years of schooling, 1–6 years and 7+ years of schooling were associated with 16 and 31% lower RRR for high frequency of self-perceived uselessness relative to low frequency, respectively. Such RRRs were only mildly attenuated yet still significant in the full model (Model VII in Table 2). Higher educational levels of spouse and father were also independently associated with reduced RRR for reporting high frequency of self-perceived uselessness relative to low frequency, but such associations were weaker compared to the respondent’s own educational level. When predicting risk of moderate frequency self-perceived uselessness versus low frequency, these RRRs were slightly attenuated (Table 3).

Living in an urban area, white-collar occupation, economic independence and good family economic condition were associated with 12–37% lower RRR for high frequency of self-perceived uselessness relative to low frequency, compared to counterparts with lower levels of resources. The reduced risk ratios for economic independence and good family economic status were moderately attenuated yet still significant in the full model while the urban residence and white collar occupation effects remained stable. This is also the case in Models II and VII of Table 3 when comparing moderate with low frequency of self-perceived uselessness.

#### Risk of self-perceived uselessness was lower in supportive and culturally traditional social environments

Results in Model III in Table 2 reveal that as a component of social environment, family/social support factors were significantly associated with self-perceived uselessness. Specifically, married older adults had a decreased RRR for high frequency of self-perceived uselessness relative to low frequency by 18% compared to unmarried counterparts. Compared to having a family member as the most frequently contacted person, having a friend/relative and having no one to contact were associated with 19 and 80% higher RRR for high frequency of self-perceived uselessness relative to low frequency, respectively. Results for the most trusted person were marginally significant. Compared to having a family member as the most helpful person, having a friend/relative as the most helpful person or having no one to ask for help was associated with 26% or 22% higher RRR for reporting high frequency of self-perceived uselessness relative to low frequency, respectively. Having available community-based services for social activities and entertainment, but not for care, was associated with 24% lower RRR for reporting high frequency of uselessness relative to low frequency. However, most of these RRRs were not significant when all other sets of factors were simultaneously controlled for in the model (Model VII). The findings in Model III in Table 3 are similar to those in Table 2 except that some of these variables were still significant in Table 3.

Results in Model IV represent cultural environmental factors that were associated with self-perceived uselessness. Coresidence with children was associated with 13% lower risk ratio for reporting high frequency of self-perceived uselessness relative to low frequency, compared to non-coresidence with children. Concordant coresidence (respondent wants to live with children and does live with children) was associated with 11% lower RRR for high frequency of self-perceived uselessness relative to low frequency, compared to those who did not fulfill their expectation of coresidence or were institutionalized (discordance). Giving financial and instrumental support to children was associated with 38% lower RRR for high frequency of self-perceived uselessness relative to low frequency, compared to those who did not provide for children. Interestingly, receiving financial and instrumental support from children was associated with greater RRR for high frequency of self-perceived uselessness relative to low frequency in Model IV, but this upward financial transfer was not significant in the full model. The RRRs of moderate frequency relative to low frequency in Table 3 were similar to those for high frequency relative to low frequency.

#### Good behaviors were associated with reduced risk of self-perceived uselessness

Good health behaviors were associated with lower risk of high or moderate frequency of self-perceived uselessness (Model V in Tables 2 and 3), independent of all other study factors (Model VII in Tables 2 and 3). Specifically, current consumption of alcohol, regular exercise, participation in leisure activities and social participation were associated with 18–54% lower risk ratio for reporting high frequency of self-perceived uselessness relative to low frequency (Model V in Table 2) while smoking was associated with 10% higher risk ratio for high frequency versus low frequency; with one exception for current smoking, these RRRs were still significant in the full model despite attenuated associations. Slightly weaker associations were found for these health behaviors in the case of moderate frequency versus low frequency.

#### Health conditions were most strongly related to self-perceived uselessness

Health conditions were all significantly associated with self-perceived uselessness (Model VI in Tables 2 and 3), even when controlling for all other factors in the REHAB model (Model VII in two tables). ADL and IADL disability, cognitive impairment and having 1+ chronic disease conditions were associated with increased RRR for high frequency of self-perceived uselessness relative to low frequency by 37–120% (Model VI in Table 2), and increased RRR of moderate frequency relative to low frequency by 12–57% (Model VI in Table 3). These RRRs were only attenuated to 22–96% (Table 2) and to 21–52% (Table 3) in the full model. Loneliness was associated with 7 times higher RRR for high frequency of self-perceived uselessness relative to low frequency (Table 2) and 2 times higher risk ratio for having moderate frequency relative to low frequency (Table 3), while high joyfulness reduced RRRs in both cases by half. These effects were only mildly weakened in the full model in both cases.

#### No clear trend over time in self-perceived uselessness

The year of survey was also significant in some cases, yet without a clear trend over time. Overall, the respondents in the 2014 wave had 21–71% greater RRR for high frequency of self-perceived uselessness relative to low frequency, compared to the 2005 wave. No difference was found for the other waves compared to the 2005 wave. However, in the case of moderate frequency versus low frequency, respondents in the 2014 wave had a 9–32% higher RRR than those in the 2005 wave and the 2008 wave tended to be associated with 5–18% lower RRR for high/moderate frequency of self-perceived uselessness compared to the 2005 wave. The sample strategy was slightly different between waves, so verification of such trends deserves a closer analysis.

## Discussion

Self-perceived uselessness, i.e., individual assessment or perception about one’s usefulness to others at older ages, is a social process [1–5, 58] that can be influenced by several types of factors. Based on a unique very large multiwave nationally representative dataset of older adults in China, the present study developed the multidimensional REHAB framework to examine factors that could be associated with self-perceived uselessness. To our knowledge, the present study is among the first to address calls to systematically examine predictors of self-perceived uselessness [18, 20, 76]. Overall, we found that a wide range of variables within the factors of socioeconomic resources (R), environments (E), health (H), fixed attributes (A) and behaviors (B) were associated with self-perceived uselessness. Specifically, high and moderate frequencies of self-perceived uselessness were more likely among individuals who were older, women, Han ethnicity, less optimistic, less self-controlled, in poor health and those who had fewer social supports, fewer resources and unhealthy behaviors. Cultural factors such as coresidence with children and giving children instrumental support were associated with lower frequency of self-perceived uselessness.

One unique finding is the relationship between fixed attribute age and self-perceived uselessness. We found that older age was associated with greater relative risk ratio (RRR) for high or moderate frequency of self-perceived uselessness relative to low frequency, which is in line with many previous studies [47, 49, 59–61]. The finding is justifiable because at older ages, health tends to decline and activities tend to decrease, leading to diminished opportunities to help others [48]. Because the CLHLS dataset had a reasonable sample of oldest-old adults, we were able to show that the RRRs became stable after age 90 (compared to the young-old aged 65–79). Moreover, when health condition and other factors were taken into consideration, the RRRs for older ages were reversed, indicating that with wellbeing held constant, the older the respondents were, the less frequently they felt useless. Empirical evidence indicates that those who survive to oldest-old ages are a very selected group compared to those who died or are in a poorer state of health in their cohort [49]. The long-lived persons have likely developed excellent coping skills to overcome health decline and daily challenges [52]; as a result, they may perceive any level of usefulness positively. This is especially true in a Confucian country where long-lived persons are generally respected. On the other hand, when young-old adults experience new negative events like illness, these problems can negatively impact their perceptions of usefulness in the absence of coping skills that develop over time [49]. Our findings for the fixed attribute of age are in line with one recent study that found no difference in self-perception of aging among the oldest-old aged 80 or older as compared to older adults aged 60–69 when health was controlled [51].

The second important finding is the importance of socioeconomic resources - not only the respondent’s own education, but also the education of significant others - in relation to self-perceived uselessness. We found that compared to those with no schooling, higher levels of education were associated with lower risk ratio for high and moderate frequency of self-perceived uselessness relative to low frequency. This finding is in line with many other previous studies [45, 58], but contradicts one recent study of older adults in Canada and Japan that showed either a negative association or no association between education of the respondents and their self-perception of aging [70]. This may be due to the lower overall level of educational attainment of the current cohorts of Chinese older adults. About two-thirds of the respondents in the current study were illiterate, whereas the proportion of respondents with 16 years of schooling was about 10% in the Japanese sample and 38% in the Canadian sample. We additionally found that educational attainments of significant others (spouse or father) were associated with respondents’ self-perceived uselessness, especially when other sets of factors were not present. The significant association of spouse’s education suggests that their knowledge and related attitudes and perceptions could play a role in the formation of respondents’ self-perception of uselessness at later ages [69]. The significant role of father’s education implies that parental education could also have a direct or indirect influence on one’s internalized perception of aging or usefulness from early life though old age. However, the roles of significant others diminished when all measured covariates were included, particularly due to intergenerational similarity in SES within families. In sum, every family member’s education could matter for respondents’ self-perception of uselessness, but the more proximate measure of their own education mattered most.

The third unique finding is the association between self-perceived uselessness and cultural environmental factors of coresidence and intergenerational transfer that are uniquely important in China. Coresidence with adult children and the concordance between expected and actual coresidence are associated with lower risk ratio for high or moderate frequency of self-perceived uselessness relative to low frequency. Because China is a Confucian society, having a large family and coresidence with children are considered as a tradition [93]. Most members of older generations consider family life, good intergenerational relations and coresidence with children to be the most important parts of their daily lives [42]. From the perspective of older adults, coresidence with children is important to ensure communication, contact and shared views and understanding with children, thus improving family solidarity. Coresidence with children also reflects the cultural tradition, which is important for older generations. That is why those who expect to coreside with children and fulfill that expectation have the greatest reduction in RRRs for high or moderate frequency of self-perceived uselessness compared to those whose coresidence expectation was not met. Furthermore, older parents can provide some assistance to coresiding adult children in terms of doing housework and taking care of grandchildren, which could enhance older adults’ feelings of usefulness to the family [93]. All of these processes would eventually benefit all domains of health and improve positive perceptions about aging among older adults.

A separate but related cultural norm, receiving financial and instrumental support from children, was associated with greater RRR for high frequency of self-perceived uselessness relative to low frequency. This seemingly counterintuitive finding is interpretable. Needing financial or instrumental support from children may indicate difficulties in older adults’ financial condition, poor health or other needs. As such, older adults may interpret receipt of transfers as a family burden [57, 104] and reinforce negative perceptions about their usefulness to the family. Taken together with the coresidence patterns, we argue that emotional support of family members may be more important in influencing older adults’ perceptions about their usefulness than financial or instrumental support. By contrast, providing financial and instrumental support to children was associated with less frequent self-perceived uselessness. This is likely because actively and capably helping children could increase older adults’ self-esteem and their perception of their value to family members [93, 105]. Additionally, providing support to children represents frequent contact with family members that can help to avoid social isolation, loneliness and unhealthy behaviors [58]. In sum, our findings related to cultural components imply that culturally normative family support is important to the formation of self-perceived usefulness in old age, which further supports the importance of family members as central forms of social support [58].

Several other fixed attributes were important. For example, we found that women’s greater RRRs for high frequency of self-perceived uselessness disappeared when health and all other factors were modeled. This is consistent with several previous studies [47, 51], and is likely influenced by their traditional gender roles and their poor health compared to men [47]. Compared to respondents of minority ethnicity, Han older adults were more likely to report high frequency of self-perceived uselessness relative to low frequency, but there was no ethnic difference between moderate and low frequency. The results for optimism and self-control are expected and consistent with the literature because optimism indicates that one is open-minded, hopeful and secure about the future, which could help one to effectively cope with adversities and conflicts in daily life [58, 106], and because self-control enables one to be actively engaged in health-promoting behaviors, which in turn develop a positive perception of aging [58].

Resources other than education were also important, including urban residence, white collar occupation, economic independence and good economic condition. This is possibly because the non-education resources increase quality of life. If people feel good about their life and living conditions at older ages, they may be less likely to see themselves as useless in old age [69]. Older adults with more resources can also afford services and products and modifications to allow them to continue to contribute despite setbacks like poor health [58], and enjoy better services that could help them overcome difficulties or adversities faced in daily life. These results are in line with previous findings that individuals who were well educated and had lower levels of economic hardship were significantly more likely to report greater levels of positive beliefs about aging [46]. Overall, one’s self-perception of aging is closely linked with resources available to that person [68]. Individuals with more resources are more likely to have positive attitudes, views and perceptions about aging because they have more opportunities and expectations [69].

Associations between good health behaviors and lower likelihood of self-perceived uselessness, independent of socioeconomic resources, environments, health conditions and fixed attributes, were expected and consistent with previous studies [34, 58]. Regular involvements in or maintenance of health behaviors such as leisure activities, exercise and social engagements could simulate body functions, buffer against negative emotional or psychological distress, develop daily coping skills and increase feelings of meaningfulness [47, 48, 58, 74]. Our findings provide additional evidence emphasizing the potential role of healthy behaviors in preventing self-perceived uselessness.

Previous research also suggests that health outcomes may be the factor that most strongly predicts self-perceptions of aging [45]. Among the most common health events associated with the aging process are those pertaining to functional health and disability [45]. Physical health conditions, such as chronic disease, functional disability, sensory performance and number of sick days, may form an underlying basis for self-evaluation of aging and health status [67]. Our findings confirm that health conditions might be possibly the most pronounced predictors of self-perceived uselessness, and that loneliness and disability might be the most significant factors compared to other health outcomes. Given the subjective nature of self-perceived uselessness, however, it is important to acknowledge that self-perceptions are not only influenced by objective health indicators, but also by psychological and social factors [67].

Our findings have important policy implications. Given China’s large size and the rapid growth of the elderly population [107], the fact that the one-fifth of this population reports a high frequency of self-perceived uselessness is a great challenge for public health. Identification of factors associated with self-perceived uselessness provides a great opportunity to target interventions and influence the health and wellbeing of the elderly population. Our findings related to cultural and social support elements imply that intervention should be oriented to supporting awareness of the value of older adults, the nature of the aging process, and the importance of family support and healthy behaviors. The intervention programs should also aim to increase dialogues between generations and different groups of people, and eventually promote frequent intergenerational contacts and geographical proximity or coresidence. Findings related to behaviors suggest that it is crucial to develop volunteer programs that facilitate community-based leisure and social engagements to promote and improve healthy behaviors associated with low frequency of self-perceived uselessness. Findings on resources and health imply that policies to support those with limited resources and poor health are also key to improve older adults’ self-perceived usefulness. The United Nations Sustainable Development Goals set for years of 2016–2030 have provided us a global context to address aging issues. The theme of the International Day of Older Persons for the year of 2016 is “taking a stand against ageism” [108]. One purpose is to draw global attention to challenging negative perceptions about aging. We hope that programs and events like these, which are consistent with the findings of this study, will influence the Chinese Government to better address self-perception of aging.

This study has the following limitations. First, self-perceived uselessness was measured by a single item. Multi-item measures of uselessness would provide a more complete reflection of the concept of uselessness [11], but may be difficult to implement in large-scale epidemiological studies. We encourage additional studies to investigate more sophisticated, positive/negative, and/or domain-specific constructs of self-perceived uselessness—and self-perceived aging more generally— to better understand mechanisms for successful aging [1, 109]. Second, we did not examine whether there is an association between changes in self-perceived uselessness and subsequent successful aging. Although previous studies showed that self-perceived uselessness is relatively stable [2, 11], changes are still frequent [1]. It would be interesting to investigate predictors of change over a longitudinal study period. Third, the relationship between self-perceived uselessness and some behaviors and health are likely bidirectional. Like most existing studies in the field [76], we did not disentangle such bidirectional associations because it was beyond the scope of the study. More sophisticated methods such as simultaneous equation modeling or structural equation modeling may shed light on this if more waves of data are available. Fourth, in the literature, coresidence has been used either as a proxy of social support or as a cultural tradition [89–95] that is determined by many other factors such as needs and resources [91, 92]. In the present study, we considered it as a cultural tradition, which may not completely capture its broad meaning. It is still a challenge to classify coresidence into a correct category and capture its meaning in the context of cultural norms. Fifth, resource factors at the aggregated neighborhood level, such as socioeconomic development and neighborhood attributes, were not considered in the analysis due to lack of data. Because there is a documented association between these factors and self-perception of aging [45], inclusion of these interesting factors would lead to a more properly specified model. Much work remains to fully utilize the important concept of usefulness to intervene and improve the lives and health outcomes of older people as they age.

## Conclusions

Based on a unique large nationally representative dataset of older adults in contemporary China from 2005 to 2014, this study found that socioeconomic resources (R), environments (E), health (H), fixed attributes (A) and behaviors (B) were associated with self-perceived uselessness at older ages. Specifically, individuals who were younger, men, non-Han, optimistic, self-controlled, healthy and with social support, healthy behaviors and better resources were significantly less likely to report high frequency of self-perceived uselessness. Cultural factors such as coresidence with children and giving children instrumental support were also associated with lower risk of self-perceived uselessness. Our findings could inform the development of targeted public health programs that aim to promote positive self-perceptions about aging in China, and possibly in other countries.

## Abbreviations

ADL: Activities of daily living
CLHLS: Chinese Longitudinal Healthy Longevity Survey
IADL: Instrumental activities of daily living
MMSE: Mini-mental state examination
