# Erratum to: Self-perceived uselessness and associated factors among older adults in China

**DOI:** 10.1186/s12877-017-0494-4

**Published:** 2017-05-02

**Authors:** Yuan Zhao, Jessica M. Sautter, Li Qiu, Danan Gu

**Affiliations:** 1 Ginling College and School of Geography Science, Nanjing Normal University, Nanjing, China; 2 Department of Behavioral and Social Sciences, University of the Sciences, Philadelphia, PA USA; 3 Independent Researcher, New York, NY USA; 4 United Nations Population Division, Two UN Plaza, DC2-1910, New York, NY USA

## Erratum

After the publication of this work [1] five corrections were discovered in the text. The corrections are as follows:

1. In the first line of the fourth paragraph in the section, Background, the text “non-Western cultures” should be “Western cultures”.
2. In the first line of the section, Factors associated with self-perceived uselessness, the text “six sets” should be “five sets”.
3. In Row 2 of Table 1, the text “Giving money/food to children-yes” should be “Giving money/food to children-no”. But the figures in the same line should stay. The “no” category should be corresponding to 77.0.
4. In Row 3 of Table 1, the text “Giving money/food to children-no” should be “Giving money/food to children-yes”. The figures in the same line should stay. The “yes” category should be corresponding to 23.0.
5. The last sentence of the first paragraph in section, Risk of self-perceived uselessness was lower in supportive and culturally traditional social environments, “The findings in Model III in Table 3 are similar to those in Table 2 except that some of these variables were still significant in Table 3” should be “The findings in Model III in Table 3 indicate that the association between social environments and self-perceived uselessness was weakened when the moderate frequency of self-perceived uselessness was compared to the low frequency”.
